# Glutathione Metabolism in *Candida albicans* Resistant Strains to Fluconazole and Micafungin

**DOI:** 10.1371/journal.pone.0098387

**Published:** 2014-06-04

**Authors:** Bruno Maras, Letizia Angiolella, Giuseppina Mignogna, Elisabetta Vavala, Alberto Macone, Marisa Colone, Giuseppina Pitari, Annarita Stringaro, Silvestro Dupré, Anna Teresa Palamara

**Affiliations:** 1 Dipartimento di Scienze Biochimiche “A. Rossi Fanelli”, Sapienza Università di Roma, Rome, Italy; 2 Dipartimento di Sanità Pubblica e Malattie Infettive, Istituto Pasteur Fondazione Cenci Bolognetti, Sapienza Università di Roma, Rome, Italy; 3 Dipartimento di Tecnologie e Salute, Istituto Superiore di Sanità, Rome, Italy; 4 Dipartimento di Biologia di Base ed Applicata, Università degli Studi L'Aquila, L'Aquila, Italy; Institute of Microbiology, Switzerland

## Abstract

Currently available therapies for candidiasis are based on antifungal drugs belonging to azole and echinocandin families that interfere with different aspects of fungal metabolism. These drugs, beyond their specific effects, elicit also a cellular stress including an unbalance of redox state that is counteracted not only utilizing antioxidant species but also increasing the outcome export by transporters to detoxify the internal environment. These cellular actions are both based on the cytosolic concentration of reduced glutathione (GSH). In this paper we investigated the effects of two antifungal drugs fluconazole and micafungin on the redox state of the cell in *C. albicans* to understand if the resistance to these drugs is accompanied by variation of glutathione metabolism. Analyses of resistant strains showed a marked difference in glutathione contents in strains resistant to fluconazole (CO23RFLC) or micafungin (CO23RFK). In CO23RFLC, the total amount of glutathione was more than doubled with respect to CO23RFK thanks to the increased activity of γ-glutamilcysteine synthetase, the key enzyme involved in GSH synthesis. We demonstrated that the GSH increase in CO23RFLC conferred to this strain a clear advantage in counteracting oxidative toxic agents while assignment of other roles, such as a more efficient elimination of the drug from the cell, should be considered more speculative. As far as MCFG resistance is concerned, from our data a role of glutathione metabolism in supporting this condition is not evident. Overall our data indicate that glutathione metabolism is differently affected in the two resistant strains and that glutathione system may play an important role in the global organization of *C.albicans* cells for resistance to fluconazole. Such scenario may pave the way to hypothesize the use of oxidant drugs or inhibitors able to deplete reduced glutathione level as a novel approach, for counteracting the resistance to this specific antifungal drug.

## Introduction

*Candida albicans* is the most important cause of fungal infection in humans, especially in immunocompromised patients [1], [2], [3]. Currently available therapies consist of antifungal drugs belonging to azole and echinocandin families that interfere with different aspects of fungal metabolism. The raise of resistant strains to these drugs may account for the dramatic increase in the incidence of nosocomial bloodstream candidiasis found in recent years [4], [5], [6]. These drugs, beyond their specific effects, elicit also a cellular stress including an unbalance of redox state [7] that is counteracted not only utilizing antioxidant species but also increasing the outcome export by transporters to detoxify the internal environment [8], [9]. The buffering of antioxidant species can be accomplished by reduced glutathione (GSH), that is also required for phase II detoxification in which endogenous and exogenous toxic metabolites are conjugated to GSH for their removal [10], [11]. Upon oxidation, GSH forms a structure made up by two glutathione molecules linked by a disulphide bridge (GSSG) that is enzimatically reconverted to the reduced species by glutathione reductase, a NADPH dependent enzyme. GSH plays a central role in a multitude of biochemical processes and perturbation in its homeostasis is implicated in the etiology and progression of several diseases. GSH is synthesized intracellularly from its amino acid precursors by the ATP-requiring cytosolic enzymes γ-glutamylcysteine synthetase (GSH1) and by GSH synthetase (GSH2). After its synthesis, GSH is delivered to other intracellular compartments through GSH transporters. The detoxifying action of GSH requires the involvement of two enzymatic families: glutathione peroxidase and glutathione transferase. The former dismutates peroxides (e.g. H_2_O_2_ and lipid peroxides) while the latter conjugates GSH with toxic metabolites for their efflux through transporters.

The success of *C. albicans* as a pathogen is partly due to its resistance to oxidative stresses and to other environmental insults [12], [13] comprising antifungal drugs. However, monitoring of GSH and GSSG levels in *C. albicans* upon drug treatment is still scant and in particular it is not known if resistant cells succeed better in counteracting drug stress by increasing GSH concentration. If this were the case, also removal of antifungal drugs, requiring GSH conjugation for their export, would be facilitated. In yeast, GSH transporters, that mediate export of GSH-conjugated substances out of the cell, involve ATP-binding cassette (ABC) membrane transporters [9]. Multiple drug resistance in *C. albicans* is associated with overproduction of ABC transporters or major facilitator (MFS) superfamilies. These transporters have been associated with azoles efflux but not with echinocandins whose outcome system has not yet been determined [9], [14]. Overall these data prompted us to investigate whether the resistance of *C. albicans* to different antifungal drugs such as fluconazole (FLC) and micafungin (MCFG), belonging to azole and echinocandin families respectively, is related to modifications of the cell redox state, focusing on glutathione metabolism. To this aim, GSH and GSSG levels and activity of enzymes involved in GSH metabolism have been analyzed in both sensitive and resistant strains treated or not with the two antifungal drugs. Our data demonstrate that intracellular glutathione levels are significantly increased in FLC resistant cells suggesting that glutathione may play a role in increasing the ability of *C. albicans* cells to survive to this drug.

## Materials and Methods

### Organisms and growth conditions

*C. albicans* strains from type collection and clinical isolates of the fungus were used throughout the study. The CO23strain was isolated from a subject with vulvovaginal candidiasis. Resistance was acquired by growth in stepwise-increasing of FLC or MCFG concentrations. The MICs values for FLC and MCFG were respectively CO23 (0,25 and 0.015 µg/ml), CO23RFK, (0,25 and 4 µg/ml) and CO23RFLC (64 and 0,015 µg/ml). As expected CO23, CO23RFLC and CO23RFK strains had an identical electrophoretic kariotype, as determined by pulse-field gel electrophoresis (PFGE), thus demonstrating that there was no selection of a contaminating FLC and MCFG resistant yeast during the process of resistance acquisition [15]. Molecular analyses showed that CO23RFLC had acquired a marked increased expression of the MDR1 gene encoding for an efflux pump protein mainly responsible for the drug-resistance, due to a mutation in the transcription factor gene MRR1 [16] whereas CO23RFK had a homozygous mutation (S645Y substitution) in the FSK1 gene, responsible for the synthesis of glucan [17].

Each strain was routinely maintained on Sabouraud dextrose agar medium (SDA; Difco, Detroit, Mi) at 28°C. For experimental purpose, strains were grown on Yeast Nitrogen Base medium (YNB; Difco, Detroit, Mi) supplemented with 0.1% glucose, at 28°C. FLC or MCFG were dissolved in distilled water at a concentration of 10 mg/ml, and added to the culture medium, to a final concentration indicated for each single experiment.

Five microliters of cell concentrations (10^5^, 10^4^, 10^3^ and 10^2^) of each yeast culture were spotted onto YPD plates treated or not with H_2_O_2_ 4 mM, in presence or in absence of the GSH precursors (0,02 M glutamate, 0,02 M cysteine, 0,02 M, glycine) or with BSO (L- buthionine-sulfoximine) 5 mM. The plates were incubated at 30°C for 2 days.

### GSH and GSSG determination

To assess the influence of FLC and MCFG on glutathione intracellular contents, each strain was cultured in YNB medium at 28°C, for 24 h, using an inoculum size of approximately 10^7^ cells/ml to recover adequate amount of cytosolic material for glutathione analysis. Under these conditions, the final OD of the control culture reached a value of 1.2–1.4. The drug was added at a concentration of (5 µg/ml), in order to provoke only a slight inhibition of growth (final OD of about 1.0). For the resistant strains, FLC and MCFG concentration were 10 µg/ml and 1 µg/ml respectively.

Cytosol of the different strains was obtained by centrifugation at 1.500 *g* for 10 min at 4°C, and suspension in 0.5 M Tris/HCl buffer (pH 6.8) 1 mM EDTA. Cells were broken by homogenization with 0.45 mm glass beads in the Bead-Beater (Biospect Products Bartelsville, Okla) kept on ice bath for 20 min, with cycles of 1 min treatment. The samples were centrifuged at 1.500 *g* for 10 min and the supernatant was stored at −80°C for subsequent analyses. Proteins concentration was determined by the Lowry assay (Sigma).

Samples, each corresponding to 10^6^ cells, were treated with 200 µl of trichloroacetic acid 5%. After centrifugation, 10 µl of the supernatant were filtered through a Microcon YM-10 centrifugal filter device (Amicon), and analyzed by reversed-phase high-performance liquid chromatography on a C18 column (Atlantis 5 µm, 4,6×200 mm, Waters) as previously described [18]. Eluate was monitored by an electrochemical four-channel colorimetric sensor (ESA model 6210) at 700, 800, 900 and 1000 mV.

GSH and GSSG values were expressed as picomoles (pmol) per microgram of protein calculated from calibration curves using standard GSH and GSSH solution. Detection limits were 0.5 pmol/μl for GSH and 1.25 pmol/μl for GSSG. For each sample, two biological replicates were analyzed with three technical replicates.

### Enzymatic activity

#### Glutathione transferase (GST)

GST activity was recorded at 340 nm and at 25°C, following the conjugation reaction of 1-chloro-2,4-dinitro-benzene (CDNB) to GSH (ε_340_ = 9.6 mM^−1^ cm^−1^). Cell extracts were added to 0.1 M potassium phosphate buffer pH 6.5, containing 1 mM EDTA, 2 mM GSH and 1 mM 1-chloro-2,4-dinitrobenzene. One unit of enzyme activity was defined as 1 µmol of GSH conjugated/min at 25°C.

#### Glutathione reductase (GLR1)

GLR1 activity was spectrophotometrically measured adding tissue extracts to 0.1 M potassium phosphate buffer pH 7.4 containing 1 mM GSSG, and 0.16 mM NADPH. The rate of NADPH oxidation was monitored at 25°C (ε_340_ = 6.22 mmol/l^−1^ cm^−1^). One unit of enzyme activity was defined as 1 µmol of NADPH oxidized/min at 25°C.

#### γ-glutamylcysteine synthetase (GSH1)

GSH1 activity was determined following ADP formation using a pyruvate kinase-lactate dehydrogenase-coupled assay. Cellular extracts were added in a final volume of 1.0 ml, to 150 mM Tris HCl buffer, pH 8.2, containing 25 mM L-glutamate, 25 mM L-γ-aminobutyrate, 10 mM ATP, 10 mM phosphoenolpyruvate, 30 mM MgCl_2_, 100 mM KCl, 0.2 mM EDTA, 0.42 mM NADH, 7 IU pyruvate kinase, 12 IU lactate dehydrogenase, and the oxidation of NADH was monitored at 340 nm. One unit of enzyme activity was defined as 1 µmol of NADH oxidized/min at 25°C.

#### Glutathione peroxidase (GPX)

GPX was measured adding cell extract to 50 mmol/l potassium phosphate pH 7.0 containing 1 mM EDTA, 1.5 mM sodium azide, 0.15 IU of purified glutathione reductase from bakers' yeast, 0.45 mM GSH, 0.2 mM NADPH, 1.2 mM cumene hydroperoxide One unit of GPX was defined as the amount of enzyme which produces 1 µmol of GSSG (oxidized glutathione)/min at 25°C.

### RNA extraction and real-time PCR

Total RNA was isolated using the RNeasy Mini kit (Qiagen) following the manufacturer's protocol. The quantity and quality of isolated RNA was determined spectrophometrically (Pearl IMPLEN nanophotometer). An equal amount (1 µg) of the total RNA for each sample was used as template to generate cDNA using iScript™ cDNA Synthesis Kit (Bio-Rad). Then an aliquot of the obtained cDNA was subjected to 40 cycles of Real-time PCR amplification (95°C, 10 sec.; 57°C, 30 sec) using the iQ™ SYBR Green Supermix and LightCycler iQ™ 5 (Bio-Rad). Primers sequences of the target genes are listed in Table 1. To ensure that the primers produced a single and specific PCR amplification product, a melting curve was performed at the end of the PCR cycle. Relative quantitative evaluation was performed by the comparative DDCt method. The mean DCt obtained in control cells (3 h or 24 h) for each gene was used as calibrator, after normalization to endogenous control β-actin (ACT1). The results are presented as fold difference relative to control.

**Table 1:** Oligonucleotide primers used for RT-PCR analysis of glutathione reductase (GLR1) and γ-glutamylcysteine synthetase (GSH1) expression.

Gene	Primer Sequence (5′- 3′)	
GLR1	GTGCTGAATTAGGTACTACATC	forward
	CCTCTGATAAAGAAATGGGTTT	reverse
GSS1	TTTGAATGGTTTGACCGATT	forward
	GTCTTACCTTCACATCCTTTAG	reverse
ACT1	GACAAATGGGTAGGGTGGGAAAAC	forward
	TGTGACAGTAACATCCCAAACGAG	reverse

Primers for actin 1 (ACT1) as housekeeping gene is also reported.

### Measurement of ROS levels

ROS formation was quantified using the irreversible conversion of dihydrorhodamine 123 (DHR123, Molecular Probes). DHR123 is a lipophilic oxidation-sensitive indicator of various ROS and known to enter cells and intracellular compartments easily. It has been widely used for testing global ROS. DHR123 is converted to green fluorescent Rh123. [19].

After treatment with H_2_O_2_ (50 mM), for 20 min, samples were treated with 1 mM DHR123 for 15 min at 37°C and then washed with cold PBS and analyzed by flow cytometer (Facscan, Becton Dickinson, Mountain View, CA, USA).

Fluorescence emission was monitored through a 530 nm band-pass filter. At least 10,000 events/sample were acquired in log mode. Fluorescence intensity is calculated as mean fluorescence channel values using the CellQuest Software (Becton Dickinson).

### Statistical analyses

Results are expressed as mean ± SD for at least three separate experiments Differences between experimental groups were determined by Student's t-test. *p*-value of <0.05 were considered statistically significant

## Results

**Figure 1:**
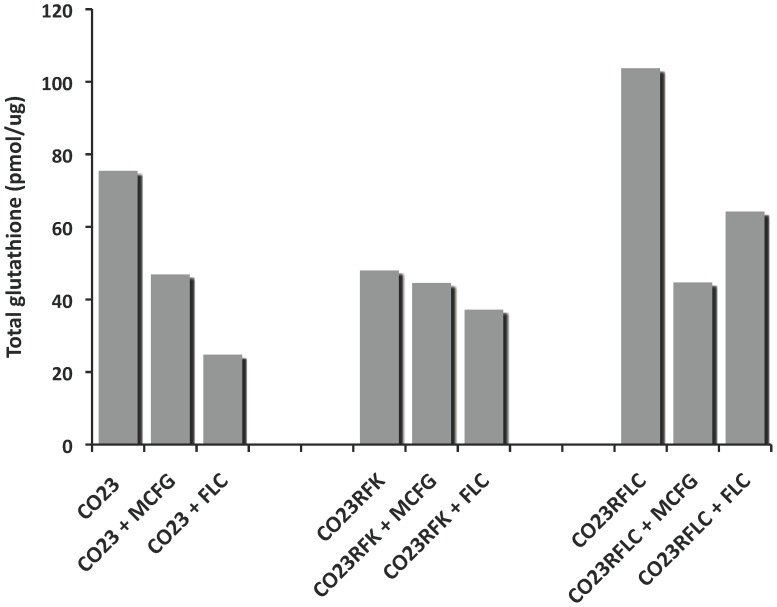
Total glutathione in CO23, CO23RFK, CO23FLC, treated and untreated with FLC or MCFG. The reported quantities were calculated using the average values for GSH and GSSG reported in Tab. 2.

In this study we monitored the intracellular amount of GSH and GSSG in the sensitive (CO23) and resistant strains to FLC or MFCG (CO23RFLC and CO23RFK) of *C. albicans*, with or without treatment with antifungal drugs. Concentrations of GSH, GSSG and the ratio between GSH and GSSG (GSH/GSSG), used as a proper value to evaluate the redox balance in these cells, are reported in Table 2. The total amount of glutathione molecules (GSH + 2GSSG) is shown in Fig. 1. When the sensitive CO23 strain was treated with MCFG or FLC, a general decrease in the GSH/GSSG ratio was observed, indicating that both these drugs induced an oxidative stress. However, total glutathione showed that the use of this peptide was different since in CO23 strain treated with FLC this value was halved with respect to the MFCG treated indicating a depletion of the glutathione pool.

**Table 2:** Glutathione amount in sensitive and resistant strains, treated and untreated either with FLC or MCFG, expressed as picomoles of glutathione for micrograms of proteins.

	GSH pmol/μg	GSSG pmol/μg	GSH/GSSG
**CO23**	68.0±3.2	3.7±0.02	18.4
**CO23 + MCFG**	18.7±2.0	14.1±0.8	1.3
**CO23 + FLC**	17.2±0.8	3.8±0.5	4.5
**CO23RFK**	42.8±3.0	2.6±0.02	16.5
**CO23RFLC**	96.9±11.4	3.4±0.08	28.5
**CO23RFK + MCFG**	38.5±1.1	3.0±0.08	12.8
**CO23RFLC + MCFG**	22.9±0.6	10.9±1.0	2.1
**CO23RFK + FLC**	30.0±4.2	3.6±0.5	8.3
**CO23RFLC + FLC**	58.6±1.6	2.8±0.2	20.9

Total glutathione is expressed as sum of the average values.

When each resistant population was treated with MCFG, CO23RFK cells showed a moderate decrease of GSH/GSSG ratio indicating a slight oxidative stress while CO23RFLC cells were dramatically affected in the their oxidative state. The treatment with FLC showed a noteworthy different behaviour between the two resistant strains. In the case of CO23RFK, the GSH/GSSG ratio dropped to a level slight higher than that of the wild-type cells treated with FLC. On the opposite, no increase of oxidative stress was observed in CO23RFLC.

Considering CO23FLC strain with respect to the sensitive one we observed an increase of intracellular level of GSH that may be due either to a stimulated biosynthesis of this compound or to a major activity of glutathione reductase able to revert the GSSG form to GSH.

To obtain a complete view of glutathione metabolism in each strain, using the same experimental panel, we examined the enzymatic activity of proteins involved in synthesis, reduction, conjugation of GSH along with the glutathione peroxidase activity that indicates the channelling of GSH towards the elimination of peroxide species produced by the oxidative stress. Specific activity of glutathione transferase (GST), glutathione peroxidase (GPX), glutathione reductase (GLR1) and γ-glutamylcysteine synthetase (GSH1), measured in the analyzed cell cultures are reported in Table 3. The most evident result was that in CO23FLC and in CO23RFLC treated with FLC cells, the activity of GSH1 dramatically increased indicating an enhanced biosynthesis of GSH. GLR1 activity markedly increased in sensitive strain treated with both antimycotics probably following the increase of the GSSG substrate. The 10-fold increase of GLR1 activity in CO23FLC may contribute to the high level of GSH along with the increased GSH1 activity. No significant differences were found for GPX and GST activities with the exception of an increase of GST activity in CO23FLC treated with FLC.

**Table 3:** Enzymatic activity of enzymes involved in glutathione metabolism assayed in sensitive and resistant strains treated and untreated either with FLC or MCFG.

	GSH tranferase mU/mg prot	GSH peroxidase mU/mg prot	GSSG reductase mU/mg prot	γ-glutamylcysteine synthetase mU/mg prot
**CO23**	0.81±0.2	5±0,8	15±2	140±14
**CO23 + MCFG**	0.72±0.2	7.3±1,1	109±5	137±15
**CO23 + FLC**	0.65±0.1	1.5±0.6	119±5	286±20
**CO23RFK**	0.73±0.2	8.40±0.8	10.9±1,2	122±20
**CO23RFLC**	2.03±0.5	1.66±0.7	158±10	431±18
**CO23RFK +MCFG**	0.66±0.1	5.3±0,9	38.4±2.4	116±15
**CO23RFLC + MCFG**	0.72±0.2	2.9±0.7	15±2,3	288±22
**CO23RFK + FLC**	1.02±0.2	1.7±0.5	19.5±6,4	281±12
**CO23RFLC + FLC**	13.5±0.7	6.9±0.9	63.6±8,3	662±19

**Figure 2:**
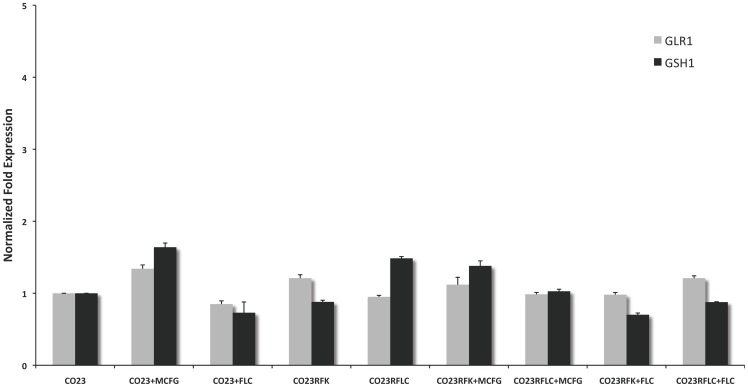
Expression levels of *C. albicans* GLR1 and GSH1 genes determined by real-time PCR. The expression level of the gene in the wild-type (CO23) or resistant (CO23RFK and CO23RFLC) strains, treated or untreated with FLC or MCFG, are represented as n-fold increase or decrease relative to the level of the untreated control strain (CO23). All bars represent mean±SD.

RT-PCR experiments were performed to assess if the increase of GLR1 and GSH1 activity might be dependent on a higher expression of these proteins. Results (Fig. 2) failed to detect any significant differences in GLR1 and GSH1 expression. Although increased GLR1 activity may be rationalized considering the increase of the GSSG substrate, the reason of the enhanced activity of GSH1 in CO23RFLC strain remains still unexplained (see Discussion).

**Figure 3:**
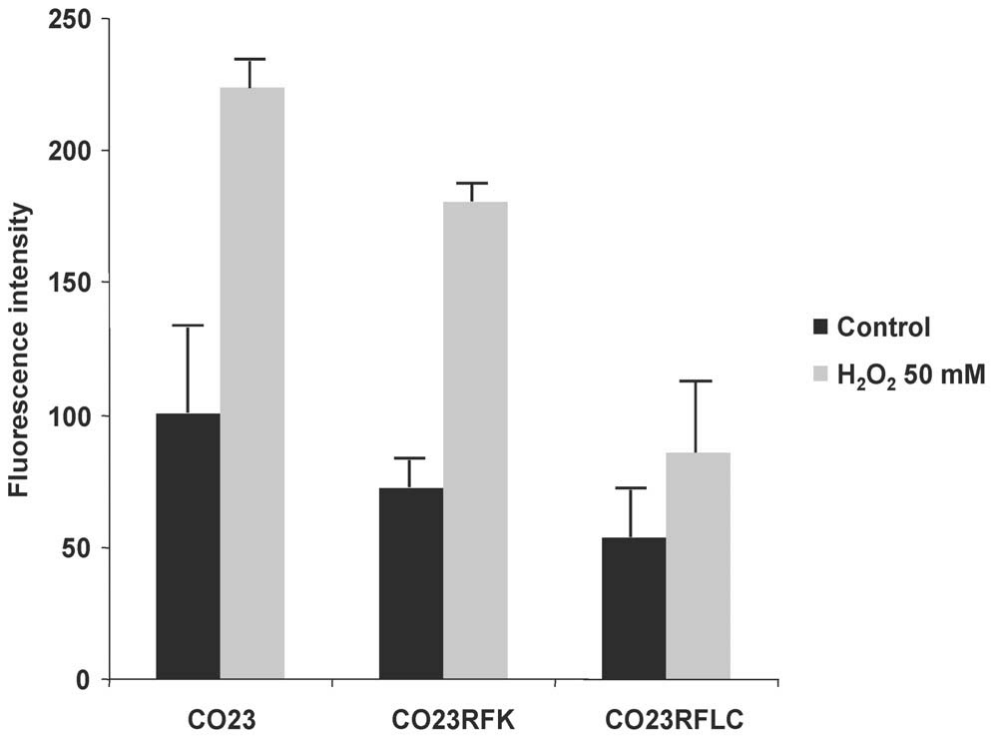
ROS production in sensitive and resistant strains. The intracellular ROS contents is evaluated by flow cytometry using DHR123 staining. Fluorescence intensity is calculated as mean fluorescence channel. All bars represent mean±SD.

To examine if the increment of GSH conferred to CO23RFLC a higher protection against oxidative stress, CO23, CO23RFK and CO23RFLC were treated with H_2_O_2_. Results reported in Fig. 3 show that, the amount of ROS was significantly lower in CO23RFLC than that found in the others strains, confirming that the resistance to FLC involved also a protective role against ROS.

**Figure 4:**
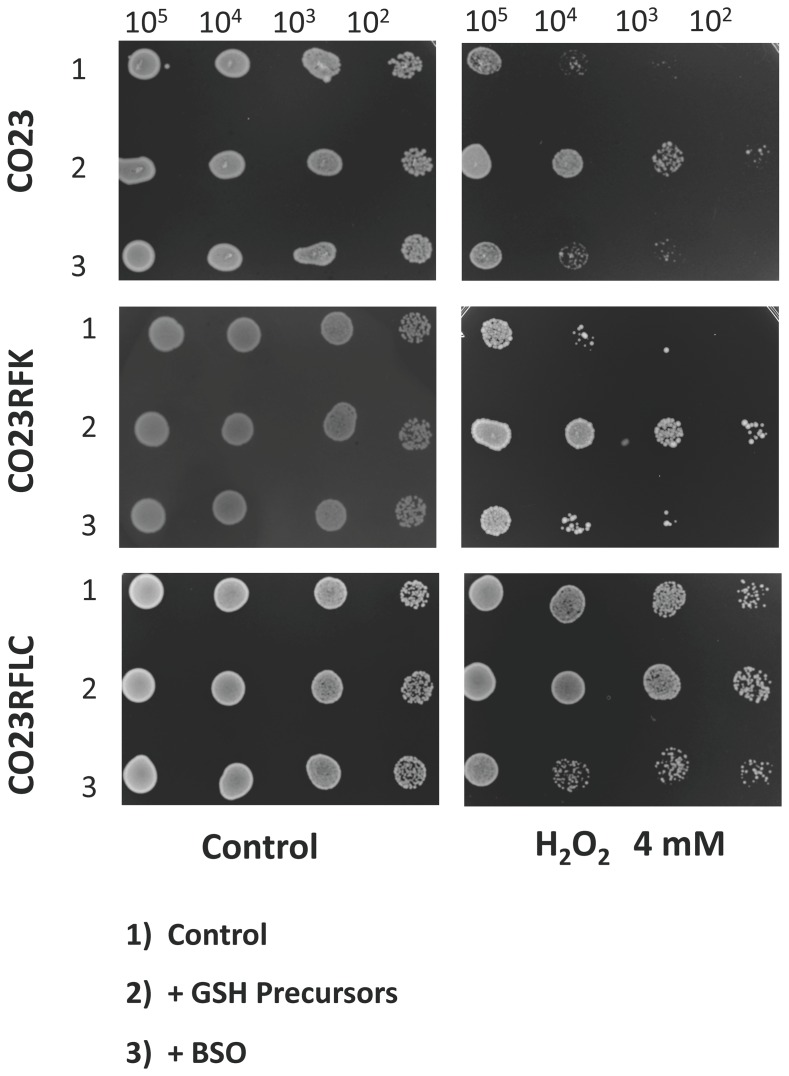
Analysis of oxidative effects in sensitive, CO23MCFG and CO23RFLC strains of *C. albicans*. Approximately 10^5^ cells of CO23, CO23MCFG and CO23RFLC strains were serially diluted and spotted onto YPD plates treated or not with 4 mM H_2_O_2_ in absence (1) or in presence of GSH precursors 0.02 M glutamate, 0.02 M cysteine, 0.02 M, glycine (2) or with 5 mM BSO (L-buthionine-sulfoximine) (3).

To further confirm that GSH increase in CO23RFLC strain was the cause of ROS protection, CO23, CO23MCFG and CO23RFLC strains were incubated in the presence of GSH precursors, glutamate, cysteine and glycine (Fig. 4). After H_2_O_2_ treatment, CO23 strain was more able to counteract the oxidative stress indicating that these amino acid precursors helped CO23 against oxidants probably increasing GSH synthesis. CO23MCFG showed a behaviour similar to that of the sensitive strain. CO23RFLC was able to counteract, either in the presence or in the absence of GSH precursors, H_2_O_2_ injury in a similar manner. However, when this strain was treated with BSO, an inhibitor of GSH synthesis, its ability to counteract the oxidative stress dropped, demonstrating that an active GSH synthesis was the cause of CO23FLC viability in the oxidative environment.

## Discussion

In this paper we investigated the effects of two antifungal drugs against *C. albicans* on the redox state of the cell, to understand if the resistance to these drugs may be accompanied by variation of glutathione metabolism. Our results show that when sensitive cells were treated with each drug, an oxidative stress took place as assessed by the decrease of the GSH/GSSG ratio demonstrating a general unspecific toxic effect of both antifungal drugs on redox balance. However, MCFG diminished GSH/GSSG ratio increasing GSSG concentration while FLC decreased the ratio mainly lowering GSH concentration resulting in a halved quantity of total glutathione. This suggests that upon FLC treatment, GSH is utilized either for the modification of proteins (glutathionylation) or it is conjugated and exported out the cell and thus no more detectable by our experimental setup.

Analyses of resistant strains showed a marked difference between CO23RFK and CO23RFLC in glutathione content. In CO23RFLC the total amount of glutathione was more that doubled with respect to CO23RFK. This increase was due to an enhanced activity of GSH1, the key enzyme in glutathione synthesis, observed both in CO23RFLC and in CO23RFLC treated with FLC. The fact that RT-PCR failed to reveal an enhanced RNA synthesis for this enzyme (Fig. 2) prompted us to conclude that GSH1 specific activity did not depend on an over expression of this protein and that other mechanisms may be involved in such a phenomenon. Regulation of the homologous γ-glutamylcysteine synthetase activity in mammals may provide help to ask this question. Mammalian enzyme is a heterodimer made up of a catalytic and a regulatory subunit able to modulate enzyme activity. Regulation is achieved also by post-translational modifications (PTMs) as phosphorylation or myristoylation and by allosteric effectors like NADPH [20]. The human catalytic subunit shares 37% identity with GSH1 of *C. albicans* indicating a clear homology between these two enzymes. On the contrary, search in Candida Genome Database with BLAST using the sequence of the human regulatory subunit failed to detect any similarities indicating that the regulation *C. albicans* enzyme is not dependent from a homologous regulatory subunit. This situation is a conundrum since homology with human regulatory subunit has been found for a great number of fungi, in particular with those from genus Aspergillus and Penicillium, ascomycetes like *C. albicans* but situated in divergent evolutive lines since the former are pluricellular organism while the latter are unicellular ones. In this frame, we speculate that the observed enhanced GSH1 activity may reside either on a PTM or on an allosteric modulation of the enzyme. Since in CO23RFLC the increased GSH concentration seems to be constitutive, a permanent covalent modification of GSH1 is likely to be the cause of this enhanced cellular concentration.

This GSH increase in CO23RFLC confers to this strain a clear advantage in counteracting oxidative toxic agents (Fig. 3) as further demonstrated by CO23, CO23MCFG and CO23RFLC strains treated with GSH precursors in presence and in absence of GSH synthesis inhibitor (Fig. 4). It should be underlined that GSH1 activity is essential to maintain the vital state of C. *glabrata* lacking transporters for exogenous glutathione and is essential for virulence in *C. albicans* [21], [22].

Assignment of other roles, beyond the anti-oxidative ones, to the GSH enhancement in the resistance to FLC should be considered more speculative. We can tentatively suppose that GSH, in this strain, may have a role in the detoxification of the cell from FLC. The increase of glutathione transferase activity in CO23FLC and markedly in CO23FLC treated with FLC focuses the attention towards a more efficient conjugation of GSH. Furthermore, in sensitive strain treated with FLC, total glutathione is markedly decreased and this evidence may be explained considering an efflux from the cell. If these observations, may be correlated with the efflux of this drug by the multidrug transporters, directly or indirectly, and in particular by MDR1, remains to be established.

Scant information is available on multidrug-transporter in Candida and intracellular glutathione. In a recent paper [23] it was reported that farnesol toxicity, leading to apoptosis in *Candida albicans*, was increased when CDR1 transporter was up-regulated. Apoptosis was due to a decrease in intracellular GSH level since farnesol is exported from the cell through CDR1 after conjugation with glutathione. Addition of exogenous glutathione maintained cell viability.

As far as MCFG resistance is concerned, from our data it is not evident a role of glutathione metabolism in supporting this condition. Indeed, it is not still clear what efflux systems are utilized by *C. albicans* cells for MCFG discharge even if it seems now clear that efflux pumps working on glutathione conjugated species utilized for FLC are not involved for this different antimycotic [15]. However it should be highlighted that when CO23RFK is treated with MCFG there was not an increase in GSSG as it usually happens when different strains are treated with this drug. The reason is not evident but our data indicate that, in CO23RFK, the toxic action of MCFG was more efficiently challenged by these cells rather than by the sensitive ones.

These data strongly suggest the idea that resistance is not only due to a mutation in a target gene but also to a more wide cellular re-organization ranging from proteomic modulations as those observed in cell wall of resistant cells [24], [25] to metabonomic variations as those related to increased levels of intracellular GSH.

In conclusion we can assess that glutathione metabolism is differently affected in the two resistant strains to the examined drugs and that in CO23RFLC, glutathione system is an important part of the overall organization of this cells for resistance.

A same role for reduced glutathione level has been claimed for cancer cell resistance to chemotherapy thus priming the use of oxidant agents as BSO to increase cell killing by antiproliferative drugs [26].

Such scenario may pave the way to hypothesize the use of oxidant drugs or inhibitors able to deplete reduced glutathione level as a novel approach, for counteracting the resistance to specific antifungal drugs.
